# Defective Intracellular Insulin/IGF-1 Signaling Elucidates the Link Between Metabolic Defect and Autoimmunity in Vitiligo

**DOI:** 10.3390/cells14080565

**Published:** 2025-04-09

**Authors:** Silvia Caputo, Federica Papaccio, Ramona Marrapodi, Gianluca Lopez, Paolo Iacovelli, Alessia Pacifico, Emilia Migliano, Carlo Cota, Anna Di Nardo, Mauro Picardo, Barbara Bellei

**Affiliations:** 1 Laboratory of Cutaneous Physiopathology and Integrated Center of Metabolomics Research, San Gallicano Dermatological Institute, IRCCS, 00144 Rome, Italy; silvia.caputo@ifo.it (S.C.); federica.papaccio@ifo.it (F.P.); ramona.marrapodi@ifo.it (R.M.); gianluca.lopez10@gmail.com (G.L.); anna.dinardo@ifo.it (A.D.N.); 2 Clinical Dermatology, Phototherapy Unit, San Gallicano Dermatological Institute, IRCCS, 00144 Rome, Italy; paolo.iacovelli@ifo.it (P.I.);; 3 Department of Plastic and Regenerative Surgery, San Gallicano Dermatological Institute, IRCCS, 00144 Rome, Italy; 4 Genetic Research, Molecular Biology and Dermatopathology Unit, San Gallicano Dermatological Institute, 00144 Rome, Italy; 5 Istituto Dermopatico dell’Immacolata (IDI-IRCCS), 00167 Rome, Italy

**Keywords:** vitiligo, metabolism, insulin, IGF-1, inflammation

## Abstract

**Background:** Vitiligo is featured by the manifestation of white maculae and primarily results from inflammatory/immune-selective aggression to melanocytes. The trigger mechanism leading to the activation of resident immune cells in the skin still lacks a molecular description. There is growing evidence linking altered mitochondrial metabolism to vitiligo, suggesting that an underlying metabolic defect may enable a direct activation of the immune system. Recent evidence demonstrated the association of vitiligo with disorders related to systemic metabolism, including insulin resistance (IR) and lipid disarrangements. However, IR, defined as a cellular defect in the insulin-mediated control of glucose metabolism, and its possible role in vitiligo pathogenesis has not been proven yet. **Methods:** In this study, we compared the Ins/IGF-1 intracellular signaling of dermal and epidermal cells isolated from non-lesional vitiligo skin to that belonging to cells obtained from healthy donors. **Results:** We demonstrated that due to the intensified glucose uptake, S6, and insulin receptor substrate 1 (IRS1) chronic phosphorylation, their inducibilities were downsized, a condition that coincides with the definition of insulin resistance at the cellular level. Correspondingly, the mitogenic and metabolic activities normally provoked by Ins/IGF-1 exposure resulted in significantly compromised vitiligo cells (*p* ≤ 0.05). Besides all the vitiligo-derived skin cells manifesting an energetic disequilibrium consisting of a low ATP, catabolic processes activation, and chronic oxidative stress, the functional consequences of this state appear amplified in the keratinocyte lineage. **Conclusion:** The presented data argue for insulin and IGF-1 resistance collocating dysfunctional glucose metabolism in the mechanisms of vitiligo pathogenesis. In vitiligo keratinocytes, the intrinsic impairment of intracellular metabolic activities, particularly when associated with stimulation with Ins/IGF-1, converges into an aberrant pro-inflammatory phenotype that may initiate immune cell recruitment.

## 1. Introduction

Vitiligo is an acquired chronic disfiguring skin disease caused by depigmentation, a consequence of the progressive loss of functional melanocytes. The clinical manifestation of vitiligo consists of white patches with variable dimensions and distribution, usually starting on the hands, face, and areas around a body’s openings and genitals. A premature whiting or greying of hairs, eyelashes, and eyebrows, as well as the involvement of mucous membranes are sometimes observed.

The pathogenic basis for this complex disease is genetic predisposition, defects in melanocyte adhesion to the epithelium, premature senescence, impaired renewal, and altered metabolic cellular activities linked to oxidative stress, which culminate in inflammatory/immune aggression against melanocytes [1,2,3,4,5]. The presence of circulating melanocyte-specific antibodies supports the autoimmune/inflammatory nature of vitiligo, the identification of many DNA sequence variants in the genes implicated in T-cell regulation, and the conspicuous immune cell infiltration in the margin of actively depigmenting skin [5,6]. However, until now no exact description of how intrinsic defects trigger an immune response has been proposed. In vitiligo, macrophages, natural killer (NK) cells, and dendritic cells (DCs) infiltrate depigmented skin during the active phase and to a minor extent, normal-appearing skin [7]. Both high levels of CD8^+^ cytotoxic lymphocytes and the related cytokines have been found on the margins of active lesions [8,9]. The recruitment of CD8^+^ T cells to vitiligo is largely dependent on locally produced IFN-γ and its target genes (C-X-C chemokine ligands) CXCL9 and CXCL10, which play an important role in the interplay of keratinocytes and lymphocytes [9,10,11]. CXCL10 can also induce apoptosis in human melanocytes in vitro via the CXCR3B receptor [12]. Moreover, like other autoimmune and chronic inflammatory diseases [13], several studies argue for a Th17 cytokine signature for vitiligo [14]. Further, vitiligo is characterized by an altered proportion and/or function of effector and regulatory T cells (Tregs) [15]. While the role of adaptative immune response in melanocyte loss has been largely documented [16], earlier events involving innate immunity have not been fully elucidated. A transcriptomic analysis revealed innate immunity activation markers in the lesional and non-lesional skin of vitiligo patients. This indicates the involvement of the entire skin surface of vitiligo patients and that additional arising factors are needed for an immunological tolerance breakdown [6]. In the complex pathogenic process of vitiligo, immune system activation may be secondary to the high oxidative stress in vitiligo skin and to the intrinsic defects in melanocytes and their microenvironment [17,18]. Vitiligo patients are reported to exhibit skin and systemic oxidative stress [19]. The high level of reactive oxygen species (ROS) in vitiligo is due to their overproduction and the significantly reduced activity of antioxidant enzymes [20,21,22]. However, some recent in vivo and in vitro studies support the possible role of altered intracellular metabolic activities in priming disease onset [23,24,25]. Redox reactions are intrinsically linked to energy metabolism and mitochondria are one of the significant sources of cellular ROS. The imbalance of mitochondrial respiratory chain complexes in the epidermis causes skin inflammation [26,27], suggesting that an intrinsic metabolic defect may enable a direct activation of the immune system. We previously demonstrated that melanocytes isolated from non-lesional vitiligo skin present defects in mitochondrial metabolism, a lower ATP production compared to control melanocytes, and a compensatory overexpression of some glycolysis-related enzymes [5]. Additionally, defects in cellular metabolism have been well documented in keratinocytes and fibroblasts [28]. More recently, pieces of evidence demonstrated the association of vitiligo with disorders related to the body’s metabolism including metabolic syndromes [29,30,31,32,33,34]. Overall, these studies converge in the demonstration of a higher fasting glucose level, a higher fasting insulin level, an increased total cholesterol amount, and a higher LDL/HDL ratio in the blood of vitiligo patients [30,32,35,36]. Current results associating type II diabetes with vitiligo [35,36,37,38,39] suggest that autoimmune and non-autoimmune factors are shared components implicated in pathogenesis. Particularly, the observation that vitiligo patients frequently manifest insulin resistance argues for a subclinical prediabetic condition [40,41]. Insulin resistance may play a role in autoimmune diseases by influencing immune system function. Exploring this link could reveal new ways to manage vitiligo, as insulin resistance could potentially affect the inflammatory processes that contribute to skin depigmentation.

The term “insulin resistance” assumes different significance when referring to the clinical (systemic insulin resistance) or cellular level. Clinically, insulin resistance implies that a higher concentration of insulin is necessary to maintain normal glucose levels; thus, it manifests in elevated glucose levels in the blood, resulting in a compensatory increase in pancreatic beta-cell insulin production and hyperinsulinemia. Until the production of the hormone is not augmented, glucose utilization is limited, and cells might suffer hypoglycemia. This condition becomes significantly evident if multiple tissues acquire a reduced response to insulin. However, a tissue-restricted impairment of the insulin signaling pathway does not necessarily exhibit systemic markers [42]. Moreover, different tissues (and/or cells) vary in their dependence on insulin, energy metabolism, and glucose uptake, implying possible heterogeneous pathologic features. On a cellular level, insulin resistance is defined as the insufficient strength of insulin signaling from the insulin receptor (InsR) downstream to the final substrates, compromising numerous metabolic aspects of cellular function. Once more, distinct types of cells might present dissimilar consequences for IR.

Ins and IGF-1 act on specific tyrosine kinase receptors, i.e., the insulin receptor (IR) and the IGF-1 receptor (IGF1R), which, once engaged, elicit the activation of a cascade of the intracellular proteins involved in the regulation of gene expression, protein synthesis, cell proliferation or death, and glucose and lipid metabolism. Insulin and IGF-1 functionally activate their receptor, but can also bind and activate the other’s receptor, although with a lower affinity [43]. In states of insulin resistance, IGF-1 can regulate glucose metabolism, complementing the effects of Ins [44], and people with diabetes can respond to IGF-1 with a beneficial effect on glucose homeostasis [45]. Studies did not reveal a distinguished gene signature regulated uniquely via the IR or IGF1R using cells expressing exclusively IGF-1 or insulin receptors. Overall, it is reported that the specificity of Ins and IGF-1 is mostly due to the levels of InsR and IGFR expression and the availability of ligands in the target tissue [46]. Keratinocytes, melanocytes, and fibroblasts have functional Ins and IGF-1 receptors indicating that intracellular-dependent signaling plays a relevant role in cutaneous physiology [47]. IGF-1 is abundantly produced in the skin by fibroblasts and melanocytes, whereas only the transcript for the receptor has been detected in keratinocytes [48,49]. Even if insulin is not locally sourced in the skin, blood-contained levels of this hormone could contribute significantly to skin biology. Accordingly, the quantity of circulating Ins has been implicated in the regulation of distant pigment patterns in zebrafish, suggesting a functional lineage-specific responsiveness to this hormone could explicate an important function for skin biology [50]. On the other hand, hyperinsulinemia leads to hyperpigmentation in humans as part of the disease acanthosis nigricans [51].

Considering that the possible connection between insulin resistance and vitiligo pathogenic mechanisms still lacks an explanation, the present study aimed to investigate if defects in insulin-mediated intracellular signal transduction in dermal and epidermal cells participate in vitiligo development. Due to the overlapping functions of insulin (Ins) and insulin-like growth factors (IGFs) and the promiscuity of the corresponding receptors’ binding, Ins and IGF-1 were both included.

## 2. Material and Methods

### 2.1. Ethical Statement

This study was conducted according to the guidelines of the Declaration of Helsinki and approved by the Ethics Committee of IFO (Istituti Regina Elena and San Gallicano).

### 2.2. Skin Biopsies and Cell Cultures

Specimens were collected from the non-lesional and non-photo-exposed skin (gluteal areas) of vitiligo subjects with non-segmental disease referred to the Vitiligo Unit of the San Gallicano Dermatological Institute–IFO–IRCCS. The gluteal area has been chosen to reduce the possible risk of anesthetic visible depigmentation after a trauma. Biopsies were collected from twenty-six vitiligo subjects (nine men and fourteen women, age range: 22–66 years; mean age 43.2 ± 12.0). At the time of enrollment, none of the subjects had a diagnosis of diabetes. Normal human skin samples were obtained from healthy volunteers subjected to plastic surgery. In this case, biopsies were collected from the abdominal area, inguinal area, breast skin, and gluteal area of twenty-five subjects (fourteen males and eighteen females, age range: 26–73 years; mean age 52.4 ± 11.4). Demographic details are summarized in Table S1. For both groups, medical records were used to ensure the absence of diabetes. After a mechanical dissection of tissue, the epidermis was separated from the dermis by digestion with dispase 0.1 mg mL^−1^ (Gibco, Thermofisher Scientific, Milan, Italy). Epidermal sheets were incubated in a solution of trypsin 0.05% and ethylenediamine tetraacetic acid 0.02% in PBS (Euroclone Ltd., Wetherby, UK) to separate cellular elements. Primary cultures of melanocytes were selectively grown in an M254 medium (Life Technologies, Monza, Italy) containing a Human Melanocytes Growth Supplement (HMGS) (Life Technologies) and antibiotics. Primary cultures of keratinocytes were selectively grown in an M154 medium (Life Technologies) supplemented with a Human Keratinocytes Growth Supplement (HKGS) (Life Technologies) plus antibiotics and Ca^2+^ at a concentration of 0.07 mM. Fibroblasts were collected from the dermises that were incubated with collagenase 0.35% (Gibco; Fisher Scientific Italia, Segrate, Milan, Italy) and then cultured in Dulbecco’s modified medium (EuroClone S.p.A., Milan, Italy) with 10% heat-inactivated fetal bovine serum (Hyclone Laboratories, South Logan, UT, USA) and antibiotics.

Commercially available THP1-Dual^TM^ cells, HEK-Blue^TM^ IL-1R cells, and all the reagents necessary for their use as indicated by the manufacturer were purchased from InvivoGen (InvivoGen, San Diego, CA, USA).

### 2.3. Cell Culture Treatments

After 24 h of starvation consisting of reducing HMGS and HKGS to 1/10 of their normal concentrations or 0.1% of FBS for the fibroblast cultures, vitiligo, and control cells were treated with 0.01 μg/mL IGF-1 or 5 μg/mL insulin (Sigma-Aldrich, Milan, Italy) for a period of five days to consider it a chronic condition.

### 2.4. Reporter Cell Lines Stimulation to Assess the Secreted Embryonic Alkaline Phosphatase (SEAP) and Luciferase Reporter Enzyme Detection

THP1-Dual cells are derived from a human monocyte cell line carrying a SEAP reporter gene controlled by NF-κB/AP-1, and an interferon regulatory factor (IRF) controlled luciferase gene. To perform the reporter cell assay, THP1-Dual cells were seeded into a 96-well cell plate and incubated with 20 μL of a conditioned medium (CM) in a total of 100 μL of the cell suspensions for 36 h before conducting the reporter assay. At the experimental endpoint, 20 μL of the cell supernatants were added to each well of a 96-well plate together with 80 μL of QUANTI-Blue Reagent (InvivoGen, San Diego, CA, USA), incubated at 37 °C for 1 h and measured in an enzyme-linked immunosorbent assay (ELISA) reader at 620 nm. The data were normalized for the number of cells corresponding to each CM. In the case of Lucia luciferase as a reporter enzyme, 20 μL of culture supernatant were transferred to a 96-well plate, but in this case, the luminometer (GloMax, Promega, Milan, Italy) automatically injected 50 μL of a QUANTI-Luc™ (InvivoGen) substrate solution per well, which was followed by an immediate luminescence measurement. The relative light units (RLUs) were normalized for the number of cells.

The HEK-Blue IL-1β cells (InvivoGen) that contained an IL-1β-sensitive reporter were used to measure the activation of the NF-κB pathway, specifically in response to IL-1β. The cells were seeded onto a 96-well plate at a density of 2.5 × 10^5^ cells/mL in 180 μL of a culture medium plus 20 μL of a keratinocyte CM. After 36 h of incubation at 37 °C, 20 μL of the cell supernatants were added to each well of a 96-well plate together with 80 μL of a QUANTI-Blue Reagent (InvivoGen), incubated at 37 °C for 1 h before reading the optical density (OD) at 620. SEAP and luciferase activities and assays were performed in duplicate.

### 2.5. Protein and Advanced Glycation End Product Determination by Sandwich ELISA

The quantity of IL6, IL-1α, -1β, IL-8, CXCL10, and TNFα in the supernatants of the control and vitiligo cells were measured by commercially available ELISA kits (Cusabio, Houston, TX, USA), according to the manufacturer’s protocols. AGEs were quantified with a Double antibody-Sandwich ELISA detection method (FineTest, Clinisciences, Rome, Italy). The results obtained were normalized for protein content in each experimental condition and were reported as picograms per 1 × 10^6^ cells.

### 2.6. Direct Total Cell Counts by Flow Cytometry

After obtaining a single cell suspension, the cells were suspended in 500 μL of PBS/0.1% FBS before being analyzed by flow cytometry. For this purpose, a volume of 100 μL was acquired in duplicate. Flow cytometry was performed on a Miltenyi MACSQuant Analyzer 10 (Miltenyi s.r.l., Milan, Italy).

### 2.7. Detection of Intracellular ROS Levels

Production of ROS was assessed with the cell permeable fluorescent dye 2′7′-dichlorodihydrofluorescein diacetate (Sigma-Aldrich). The cells were incubated with 2.5 μmol L^−1^ H_2_DCF for 30 min at 37 °C and 5% CO_2_ in a phenol red-free full-starved medium in the dark. After removing the probe solution, the cells were washed with PBS, trypsinized, centrifuged at 1000 rpm, and then resuspended in PBS. After the oxidation of H_2_DCF into fluorescent DCF by ROS, signals were measured by flow cytometry using a Miltenyi MACSQuant Analyzer 10 (Miltenyi s.r.l.).

### 2.8. Measurement of Mitochondrial Membrane Potential (ψ_m_)

We employed 5,5′,6,6′-tetrachloro-1,1′3,3′-tetraethylbenzamidazol-carboncyanine (JC-1) to assess mitochondrial membrane potential. The cells were incubated with JC-1 at a concentration of 2 μM for 30 min at 37 °C with 5% CO_2_. The cells were detached and subsequently collected by centrifugation (1000 rpm for 5 min at 4 °C) and resuspended in PBS. The double fluorescence, either as green J-monomers or as red fluorescent J-aggregates, was measured by flow cytometry using a Miltenyi MACSQuant analyzer 10 (Miltenyi s.r.l.). A higher red–green fluorescence ratio indicates a more polarized (hyperpolarized) mitochondrial inner membrane.

### 2.9. Mitochondrial Mass Measurement

The cells were stained with 0.1 μM of MitoTracker^®^ probe (ThermoFisher Scientific) for 30 min at 37 °C and 5% CO_2_ in a phenol red-free full-starved medium in the dark. After removing the probe solution, the cells were washed with PBS, trypsinized, centrifuged at 800 rpm, and resuspended in PBS. Fluorescence signals were detected by MACSQuant Analyzer 10 Flow Cytometer (Miltenyi s.r.l.)

### 2.10. ATP Measurement

The intracellular ATP concentration was measured using the ATP-determination kit (ThermoFisher Scientific, Monza, Italy). The cells were collected and immediately placed in an ice-cold passive lysis buffer (Promega, Milan, Italy) for 15 min. Cell lysates were then centrifuged at 10,000× *g* for 10 min and a supernatant was used in the assay for protein concentration determination for results normalization. A 10 μL sample was added to 90 µL of a reaction buffer in each well of a 96-well plate in the dark. Luminescence was measured using a PerkinElmer Victor3 Luminometer (PerkinElmer Italia, Milan, Italy). Each experimental sample was tested in triplicate, and the background luminescence was subtracted from the measurement before calculating the ATP concentrations using an ATP standard curve.

### 2.11. RNA Extraction and Quantitative Real-Time PCR

Total RNA was isolated by using the Aurum Total RNA Mini Kit (Bio-Rad Laboratories s.r.l., Milan, Italy). cDNA was synthesized from 1 μg of total RNA using the RevertAid First-Strand cDNA Synthesis Kit (Thermo Fisher Scientific, Monza, Italy). For a semi-quantitative real-time PCR, cDNA was amplified with a ChamQ Universal SYBR qPCR Master Mix (Vazyme Biotech, Nanjing, China) containing 25 pmol of forward and reverse primers using a CXF96 Touch Cycler (Bio-Rad Laboratories s.r.l.). All samples were tested in triplicate. The levels of gene expression were quantified by applying the 2^−ΔΔCT^ method, using β-actin as an endogenous control, and they are expressed relative to the untreated control starved cells.

### 2.12. Western Blot Analysis

Cell extracts were prepared with RIPA buffer containing proteases and phosphatase inhibitors. Equal amounts of proteins were resolved on an SDS–polyacrylamide gel, and transferred to a nitrocellulose membrane (Amersham Biosciences, Milan, Italy). To avoid running separate experiments for each protein, if the targets had relevant different molecular weights, the membranes were cut into pieces. The membranes were then treated with the following primary antibodies: rabbit pAKT-Ser473, pmTOR-Ser2421, mTOR, pS6-Ser235/236, pSer612-IRS1, pAMPK-Thr172, AMPK, LC3, pStat1-Y701 and Stat1 (Cell Signaling Technology, Leiden, The Netherlands), Glu4 (Abcam, Cambridge, UK) and mouse S6, AKT (Cell Signaling Technology, Danvers, MA, USA), and cofilin (BioRad, Hercules, CA, USA) antibodies. Horseradish peroxide-conjugated goat anti-mouse and goat anti-rabbit antibody complexes were detected by chemiluminescence (Cell Signaling). Densitometric analyses were performed with the UVITEC Mini HD9 acquisition system (Alliance UVItec Ltd., Cambridge, UK).

### 2.13. Glucose Up-Take Determnation

To evaluate the amount of glucose internalization by the vitiligo and control cells, we used an indirect method based on the spectrophotometric measurement of residual glucose in the medium 24 h after medium change. The M254 complete mediums or the low glucose DMEMs plus 10% DMEM (both with physiological glucose concentration) prepared for each experiment and maintained in the incubator with cell plates were used as internal references for each experiment. Glucose internalization was calculated for differences and reported as mg/dl after normalization for the cell number.

### 2.14. Statistical Analysis

Quantitative data were reported as mean ± standard deviation (SD). A multiple comparison analysis was performed with an ANOVA test followed by Bonferroni correction using GraphPad Prism software (version 10.4.2). The student *t*-test was used to assess statistical significance when comparing the vitiligo and healthy groups and an mRNA analysis was performed. The thresholds correspond to * *p* ≤ 0.05, ** *p* ≤ 0.01, *** *p* ≤ 0.001, and **** *p* ≤ 0.0001.

## 3. Results

### 3.1. IGF-1- and Insulin-Dependent Mitogenic Activity Is Attenuated in Vitiligo Cells

**Figure 1:**
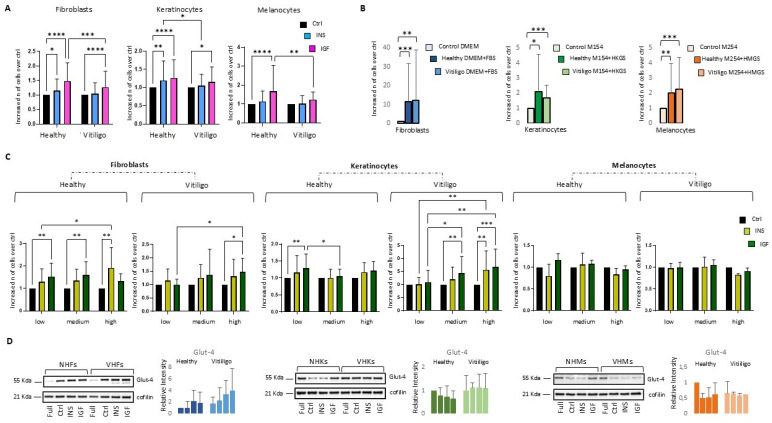
IGF-1/Ins-dependent mitogenic stimulation is attenuated in vitiligo cells. (**A**) The impact of IGF-1 and Ins on vitiligo and healthy cell proliferation was evaluated following five days of continuous treatment. Histograms report the fold-change of cell number compared to the corresponding unstimulated starved sample (Ctrl), arbitrarily indicated as 1. For statistical analysis, data were combined in histograms (NHFs *n* = 23, VHFs *n* = 18; NHKs *n* = 19, VHKs *n* = 13; NHMs *n* = 8, VHMs *n* = 6). (**B**) The proliferation rate of cells supplemented with a full medium was compared to the corresponding starved samples arbitrarily indicated as 1. The numbers of samples analyzed were the same as those reported in (**A**). (**C**) IGF-1 and Ins stimulation were combined with an increasing glucose concentration to measure their effect on cell proliferation. For statistical analysis, data from all cell cultures were combined in histograms (NHFs *n* = 12, VHFs *n* = 10; NHKs *n* = 8, VHKs *n* = 8; NHMs *n* = 4, VHMs *n* = 4), reporting the fold-change of cell number compared to the corresponding unstimulated control starved sample. * indicates *p* ≤ 0.05, ** indicates *p* ≤ 0.01, *** indicates *p* ≤ 0.001, and **** *p* ≤ 0.0001 vs. untreated cells. (**D**) Densitometric analysis and one representative Western blot for each cell type studied for the expression of Glut-4 were performed (NHFs *n* = 7, VHFs *n* = 7; NHKs *n* = 4, VHKs *n* = 4; NHMs *n* = 4, VHMs *n* = 4). Histograms report band intensity as mean ± S.D. Cofilin was included as a protein load control. Note that * indicates *p* ≤ 0.05, ** indicates *p* ≤ 0.01, *** indicates *p* ≤ 0.001, and **** *p* ≤ 0.0001.

Ins and IGFs are important regulators of energy metabolism, homeostasis, and growth. IGF-1 and Ins-induced pro-mitogenic effects have been previously demonstrated in vitro on melanocytes [47,48,49], keratinocytes [52], and fibroblast cell cultures [53]. Accordingly, IGF-1 and Ins are alternatively added to a chemically defined medium employed for the isolation and expansion of melanocyte and keratinocyte cultures, because the stimulation of the corresponding receptors plays a relevant role in the initial seeding capacity, survival, and proliferation of these types of cells [54,55]. Here, vitiligo cell growth in response to IGF-1 and Ins was assessed at concentrations routinely used for in vitro studies (0.01 μg/mL and 5 μg/mL, respectively) [55]. After 5 days of continuous treatment, the cells were prepared in the minimal medium for 24 h before stimulation. Overall, IGF-1 exerted a significant pro-mitogenic response in all the cell types tested, whereas Ins influenced, to a minor extent, keratinocyte and fibroblast proliferation and failed at achieving a significant pro-mitogenic effect on melanocytes (Figure 1A).

The results are consistent with the general findings that in mammals in vivo Ins generates mainly a metabolic response, whereas IGF-1 mediates growth-promoting effects [46]. Interestingly, both growth factors provoked a generally attenuated response in the vitiligo cells compared to the controls. Accordingly, values related to VHFs and VHKs lose statistical significance in the case of Ins stimulation. By contrast, the full medium formulations, such as the defined medium containing the complete growth factors cocktail and DMEM plus FBS, in the case of fibroblast cultures, supported cell proliferation to a similar extent in the healthy and vitiligo cells, confirming the specific deficiency of Ins/IGF-1 signaling activation (Figure 1B). Cell proliferation evoked by Ins/IGF-1 largely depends on glucose concentration and the studies combining Ins or IGF-1 supplementation with hyperglycemia reported a faster proliferation rate [56,57,58]. This implies that DMEM used for fibroblast cultures and carrying 4.5 g/L of glucose corresponding to a severe hyperglycemic condition might not be comparable to the chemically defined medium employed for epidermal cells, containing a physiological glucose level (1.0 g/L). However, other investigations reported attenuated stimulation by Ins in the presence of a glucose overload [59], indicating that cell type-specific behavior likely complicates the outcome of Ins/IGF-1 treatment in terms of cell growth. Thus, we further studied the relationship between glucose availability and proliferation in our system. Reconduction of glucose to a physiological amount (1.0 g/L) did not influence the rate of fibroblast proliferation, whereas the elevation of glucose over the standard doses used in the medium formulation evidenced that keratinocytes, melanocytes, and fibroblasts suffered hyperglycemia, suggesting a possible toxic effect due to the glucose overload (Figure S1). Surprisingly, a new set of proliferation assays evidenced that the hyperglycemic condition rescued the capacity of VHFs, and with a greater magnitude, the capacity of VHKs to proliferate in response to Ins/IGF-1, hinting that this condition in some way compensated for the disease-associated metabolic defect (Figure 1C). Of note, a significant difference between the healthy and vitiligo fibroblasts was also observed in the DMEM containing low glucose (1.0 g/L), since the NHFs maintained their capacity for faster cell proliferation due to IGF-1 exposure whereas the VHFs completely failed, confirming the necessity of a large amount of glucose. Hence, a possible explanation for the vitiligo cell’s role is anomalous necessity of glucose to supply biological activity linked to Ins/IGF-1 stimulation and to rescue the related pro-mitogenic activity. In line with this hypothesis, the increased expression of the glucose transporter Glut-4 in the vitiligo cells indicated the extended glucose requirement and cytosolic hyperglycemia (Figure 1D). Differently, the analysis of Ins/IGF-1-dependent proliferation in both healthy and pathological melanocytes in the function of glucose availability did not evidence any modification and corroborated the weak broad propensity of melanocyte lineage to react to IGFR and InsR engagement. Overall, these data indicated that a glucose-dependent metabolic defect slants keratinocytes and fibroblasts glucidic metabolic activity into hyper-functionality, suggesting a strong role in the pathogenic mechanism.

### 3.2. Molecular Impairment of IGF-1/Ins Signaling in Vitiligo Cells

**Figure 2:**
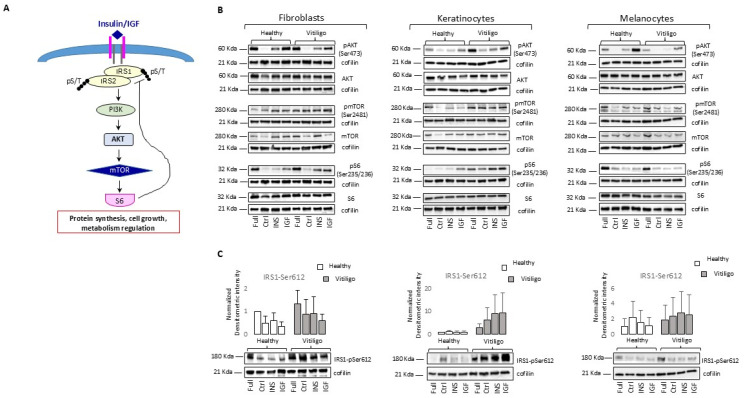
Altered IGF-1/Ins intracellular signaling in vitiligo cells. (**A**) Schematic representation of protein components involved in IGF-1/Ins signal pathway. (**B**) Western blot analysis for total and phosphorylated proteins involved in intracellular metabolic pathways activated by IGFR and InsR engagement. Representative images were presented for each cell type. (**C**) Representative Western blot images and corresponding densitometric quantification of the phosphorylation of IRS1 at serine 612. Histograms report the mean intensity ± S.D. (NHFs *n* = 10, VHFs *n* = 10; NHKs *n* = 8, VHKs *n* = 8; NHMs *n* = 4, VHMs *n* = 4). Cofilin was included as the protein load control.

Upon IGF-1 and Ins binding to the receptors, the associated tyrosine kinase activity engages intracellular insulin receptor substrates (IRS1 and IRS2), which in turn, through the stimulation of PI3K, activates AKT by phosphorylation, which results in the mammalian target of rapamycin (mTOR) switching on and enhanced glycolysis [58]. mTOR and its target p70S6K, being key regulatory proteins involved in translation and protein synthesis, function as the main signaling hub in control of cellular metabolism (Figure 2A).

By dissecting these intracellular cascade events, we observed that the phosphorylation of mTOR and its downstream target ribosomal protein S6 resulted in an unexpected higher activation in VHKs and VHFs (Figure 2B). The intermediate step involving the phosphorylation of AKT at Ser473 evidenced the divergent outcomes for keratinocytes and fibroblasts (Figure 2B). Under Ins/IGF-1 stimulation, VHFs and VHMs displayed a lower capacity to activate AKT compared to the controls, whereas VHKs displayed a more intense phosphorylation of AKT in the presence of hormones as well as when maintained in a full medium. A possible explanation for the observed dissimilarities might reside in the glucose concentration in the environment since AKT-Ser473 is linked to glucose uptake in the function of energetic needs and glucose availability. In line with this idea, a further increase in glucose concentration (25 g/L) in the DMEM mildly diminished AKT-pSer473 in the NHFs but strongly impacted this event in VHFs (Figure S2a). Similarly, both the healthy and vitiligo keratinocytes failed to phosphorylate AKT under Ins/IGF-1 stimulation in a hyperglycemic condition (Figure S2b). Correspondingly, in NHKs, VHKs, and NHFs, basal pAKT is inversely correlated to the environmental level of glucose in the medium, confirming the negative effect of glucose-dependent metabolic activities on AKT stimulation (Figure S2c,d). VHKs exacerbated the activation of AKT at all the glucose concentrations tested, which might result from a defective energetic equilibrium resembling hypoglycemia. In line with the idea that vitiligo cells perceive apparent hypoglycemia, the glucose uptake measured in the VHK and VHF cultures was higher than that of the control samples (Supplementary Figure S2e), indicating an adaptative mechanism to maintain an adequate mitochondrial fitness. On the contrary, in VHFs this modulation was compromised and pAKT phosphorylation raised in relation to the glucose amount (Figure S2c). However, in both keratinocytes and fibroblasts, the augmented pS6/S6 ratio in vitiligo cells remained unchanged. A persistent hyperphosphorylation of S6 is the recurrent marker of a vitiligo-associated metabolic impairment and demonstrates that the extracellular hyperglycemic state is differently managed by normal and pathologic cells. Several reports documented that, independent of insulin signaling, glucose activates mTOR, which in turn, has a negative feedback loop, blocking the insulin-dependent signaling pathway [59] causing insulin resistance. This resistance is characterized by the phosphorylation of the Ser^307^, Ser^612^, and Ser^318^ residues of IRS1, high levels of pS6, and a compromised insulin-induced activation of Akt. According to the molecular landscape observed and suggestive of IR, the vitiligo cells maintained a tendency of staying in a chronic hyperphosphorylated state of IRS1-Ser^612^ under Ins/IGF-1 stimulation (Figure 2C), The high basal level of IRS phosphorylation corresponds to a limited potential for further activation, which explains the blunted insulin response in the vitiligo cells. Interestingly, in the vitiligo cells, the augmented hyperphosphorylation of IRS was also evident in the starved cells. Collectively, in the vitiligo cells, the basal activation of mTOR, the high level of phosphorylated S6, and poor inducibility by insulin reflected in low Akt phosphorylation fully correspond to the description of cellular IR. However, for vitiligo, the keratinocytes attenuated InsR/IGFR intracellular signaling activation was not associated with reduced pAKT, denoting a cell type-specific feature.

### 3.3. Impairment of Insulin/IGF-1 Intracellular Metabolic Activities Exacerbate Metabolic Stress in Vitiligo Cells

**Figure 3:**
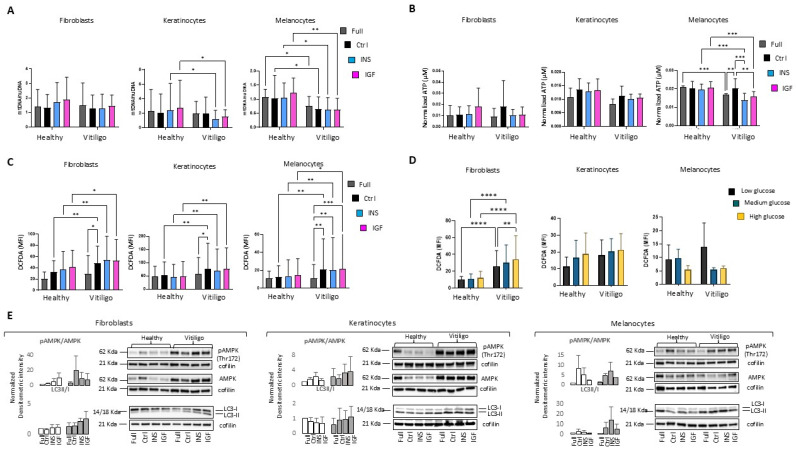
An evaluation of IGF-1, Ins, and glucose effects on the energetic state and oxidative stress. (**A**) Relative mtDNA quantification was performed on the fibroblast (NHFs *n* = 23 and VHFs *n* = 19), keratinocyte (NHKs *n* = 8 and VHKs *n* = 8), and melanocyte (NHMs *n* = 9 and VHMs *n* = 6) samples in triplicate using qPCR by amplification of mtDNA with mtDNA-specific primers and this was normalized against nuDNA amplification. The histograms present mean fold-change over the corresponding starved control ± SD. The decrease in the mtDNA/nuDNA ratio presented in the vitiligo samples is indicative of the decrease in the mtDNA copy number. (**B**) The intracellular amount of ATP normalized against the protein content was measured to compare the energetic efficiency of the healthy and vitiligo cells. The histogram report means ± SD of three independent experiments for each cell type. (**C**) The amount of intracellular ROS generated by metabolic activities confirmed intrinsic oxidative stress in the vitiligo cells (NHFs *n* = 14, VHFs *n* = 14; NHKs *n* = 8, VHKs *n* = 8; NHMs *n* = 7, VHMs *n* = 7). (**D**) The ROS generated by increasing the glucose concentration was measured with DCFA-DA as reported in c (NHFs *n* = 8, VHFs *n* = 8; NHKs *n* = 6, VHKs *n* = 6; NHMs *n* = 3, VHMs *n* = 3). (**E**) Representative Western blot images and a densitometric quantification of the pAMPK/AMPK and LC3I/II expression ratios confirmed metabolic impairment and catabolism activation (NHFs *n* = 4, VHFs *n* = 4; NHKs *n* = 4, VHKs *n* = 4; NHMs *n* = 3, VHMs *n* = 3). Cofilin was included as protein load control. Note that * indicates *p* ≤ 0.05, ** indicates *p* ≤ 0.01, *** indicates *p* ≤ 0.001, and **** indicates *p* ≤ 0.0001.

We previously demonstrated that melanocytes isolated from normally pigmented vitiligo skin have a lower mitochondrial DNA (mtDNA) copy number compared to healthy melanocytes, and that defects in mitochondrial metabolism culminate in inadequate cellular energetic function evidenced by the low level of adenosine triphosphate (ATP) and increased generation of ROS. In this study, extending the analysis to keratinocyte and fibroblast cell cultures, we confirmed that this feature is characteristic of all vitiligo cells (Figure S3a–c). Further, the depolarization of the mitochondrial membrane confirmed energetic dysfunction in the vitiligo fibroblasts and melanocytes, although an unexpected hyperpolarization was evident in the case of VHKs (Figure S3d). Interestingly, more polarized mitochondria, sometimes associated with a reduced mitochondria mass, have been reported as a specific bioenergetic alteration in the immune cells of type I and type II diabetes patients [60,61]. The quantification of mitochondria mass revealed no differences between the vitiligo and control cells (Figure S3e), whereas a tendency of a lower mtDNA/Mitotracker ratio was observed (Supplementary Figure S3f), suggesting that vitiligo mitochondria contain a reduced mtDNA copy number in enlarged mitochondria. There is an intricate interplay between IGF-1/Ins signaling and a healthy mitochondria metabolism. IGF-1 signaling stimulates mitochondrial biogenesis by increasing the mitochondrial DNA copy number in several cell types [61,62]. Here, in the healthy dermal and epidermal cells, the persistent stimulation of IGF-1 and Ins lightly augmented the mtDNA copy number in normal cells but failed at recovering the physiological mtDNA content in vitiligo cells, which remained lower in the pathological cells compared to the controls, particularly under Ins stimulation (Figure 3A).

In the healthy cells, the preservation of energetic equilibrium in the presence of IGF-1 and Ins was associated with a sustained level of intracellular ATP (Figure 3B). Furthermore, the intracellular level of ATP was faintly but constantly reduced by the addition of Ins and IGF-1 in vitiligo cells, indicating that the stimulation of receptors aggravates the intracellular energetic balance in the pathological context. Of note, the differences reached statistical significance (*p* ≤ 0.05) only in the melanocytes, probably because this type of cell presents a high energetic requirement due to the melanogenic biosynthetic process. Surprisingly, in contrast to the cell cultures maintained with the complete growth factors cocktail, the starved vitiligo keratinocytes and melanocytes did not display lower ATP levels compared to the reference samples. However, even if in this condition most of the intracellular activities are in a state of stand-by/quiescence associated with minimal energy dissipation, the vitiligo cells still displayed a significantly higher intracellular ROS content (*p* ≤ 0.01), strengthening the assumption that oxidative stress originates intrinsically, whereas energetic defects emerge in the function of the metabolic requests (Figure 3C). Of note, prolonged exposition to IGF-1 and Ins, only in the vitiligo cells, resulted in toxic ROS production, exacerbating the inherent oxidative stress. In the normal cells, particularly in the keratinocyte cultures, the ROS augmented in function of glucose concentration in the medium, whereas in the vitiligo cells the ROS levels were constantly high (Figure 3D), reinforcing the idea that the glucose perceived by vitiligo cells is chronically higher than that of the control samples. Interestingly, a different attitude emerged in melanocytes, in which augmented glucose corresponded to a lower level of ROS. A diminished cellular energy state results in the protracted activation of the AMP/ATP sensor, AMP-activated protein kinase (AMPK), in VTG cells, confirming metabolic dysfunction (Figure 3D). The physiological consequence of the energy deprivation of cells is the activation of AMPK, which limits biosynthetic activities and promotes catabolic pathways, including glucose uptake, glycolysis, fatty acid uptake, and the recycling system [63]. Our results confirmed the work of previous studies [26] reporting a constitutive enhanced expression of the well-established autophagosome marker LC3 in vitiligo cells and evidenced an additional effect attributed to exposure to Ins and IGF-1, which reflects the shift to a recycling catabolic metabolism in line with pAMPK activation.

### 3.4. Unbalanced Glucose Metabolic Pathways Cause the Overproduction of Endogenous Advanced Glycation End Products in Vitiligo Keratinocytes

**Figure 4:**
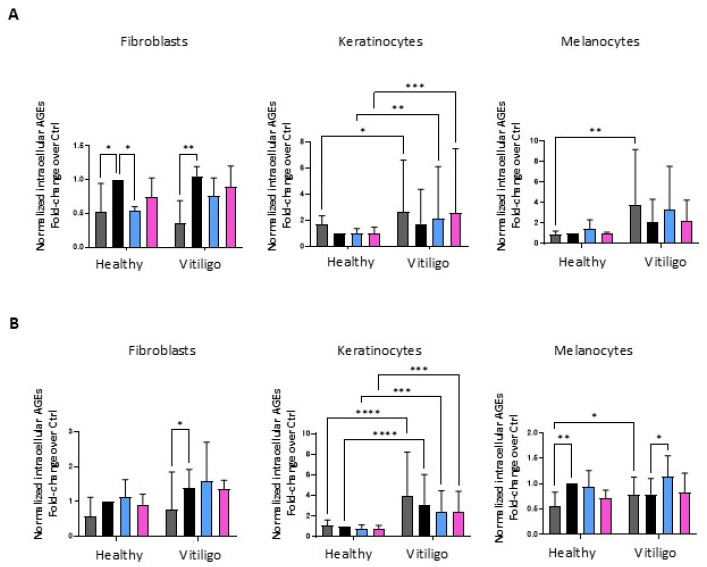
VHKs overproduce and release AGEs. Immunoenzymatic intracellular (**A**) and extracellular (**B**) quantification of AGEs in fibroblasts, keratinocytes, and melanocytes treated (or not treated) with Ins and IGF-1. Histograms represent the mean ng/mL ± SD (NHFs *n* = 3, VHFs *n* = 3; NHKs *n* = 9, VHKs *n* = 9; NHMs *n* = 5, VHMs *n* = 5). Note that * indicates *p* ≤ 0.05, ** indicates *p* ≤ 0.01, *** indicates *p* ≤ 0.001, and **** indicates *p* ≤ 0.0001.

Advanced glycation end products (AGEs) are the proteins and lipids that become glycated by a non-enzymatic reaction because of excessive free glucose exposition. Since we recently reported abnormal elevated AGEs in vitiligo patients’ blood [34], here, we analyzed these compounds in the vitiligo cell cultures. At the intracellular level, few differences were observed when comparing the accumulation of AGEs in the cytoplasm of VHFs and VHMs with the matched controls (Figure 4A), whereas, compared to the references, the patient-derived keratinocytes displayed a significantly higher level of AGEs in the cytoplasm and more importantly in the supernatant (Figure 4B), suggesting that these molecules might act as messengers in the surrounding environment.

A faster AGE formation, which represents the by-product of glucidic metabolic activities, confirmed the necessity of abnormal levels of glucose due to suboptimal metabolism efficacy in vitiligo cells. At the same time, the consequence of the adaptation to metabolic impairment implied the production and the consequent accumulation of AGEs in the extracellular milieu. This specific cell damage, mainly manifested in the keratinocyte lineage, will have a profound effect on the entire surrounding tissue, triggering oxidative stress generation and inflammation.

### 3.5. Insulin and IGF-1/Ins Stimulation Supports a Pro-Inflammatory Phenotype in Vitiligo Keratinocytes

**Figure 5:**
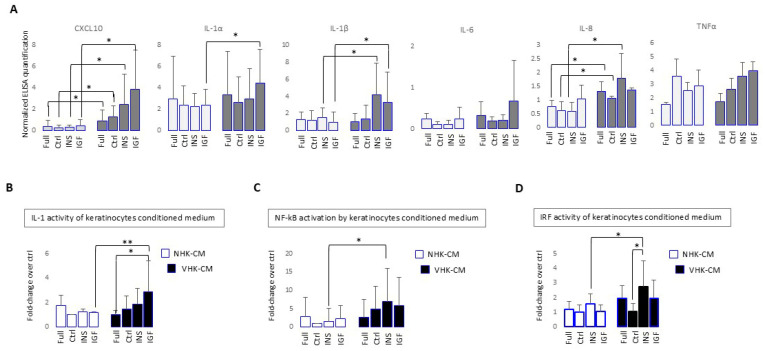
Pro-inflammatory phenotype of VHKs is exacerbated by IGF-1 and Ins stimulation. (**A**) Immunoenzymatic quantification of interleukin and chemokine production in NHKs and VHK-CM induced by each treatment regimen. Each point represents mean ± SD of at least 5 different experiments. (**B**) Receptor binding assay used to evaluate IL-1α and IL-1β activities of CM on HEK-Blu IL-1R cell line (NHKs *n* = 12, VHKs *n* = 10). Colorimetric detection at 620 nm O.D. is proportional to bioactive IL-1 (NHKs *n* = 8, VHKs *n* = 8). (**C**) Activation of NF-κB in THP-1 Dual cells in response to keratinocyte CM assessed by SEAP activity (NHKs *n* = 8, VHKs *n* = 8). (**D**) IRF pathway stimulation was measured by assessing the activity of a secreted luciferase (IRF-Lucia reporter gene), in THP-1 Dual cells treated with NHK- and VHK-CM. For (**B**–**D**), after normalization for the number of cells corresponding to the CM used, values are reported as fold-change ± SD against control (starved) NHK-CM. Note * indicates *p* ≤ 0.05, and ** indicates *p* ≤ 0.01.

Several studies reported that mitochondrial dysfunction manifested by ATP deficiency, the overproduction of ROS, and AGEs is associated with inflammatory reactions. The AGE-RAGE (AGE receptor) interaction stimulates a vast array of cell signaling pathways and transduction factors, including transcription factors, nuclear factor kappa B (NF-κB), the activating protein-1 (AP-1), signal transducers, and the activators of transcription (STAT) factors involved in inflammation. Thus, to verify if Ins and IGF-1 evoke a pro-inflammatory feature in vitiligo keratinocytes and the possible role in immunopathogenesis, we quantified the amount of several secreted interleukins that demonstrated increased quantities of CXCL10, IL-6, IL-8, IL-1α, IL1-β, and TNFα (Figure 5A).

IL-1 is a critical cytokine involved in controlling innate and adaptive immunities. To fully achieve its activity, IL-1β and to a lesser extent IL-1α, require proteolytic cleavage by inflammatory caspases during inflammasome activation, in addition to an extracellular release [64]. Thus, to confirm IL-1 functional activation we used HEK-Blue IL-1R cells carrying the IL-1-sensitive SEAP reported gene. The data reported in Figure 5B show the sustained activation of IL-1R by the vitiligo-derived supernatants compared to control samples, which specifically worsened in the case of keratinocytes maintained under prolonged stimulation with IGF-1 and insulin. The activation of NF-κB was further demonstrated using another reporter cell line; the THP-1 Dual monocytes, having SEAP reporter gene and driven by an IFN-β minimal promoter, fused to five copies of the NF-κB consensus transcriptional response element (Figure 5C). The same reporter cell line, also carrying the Lucia reporter gene driven by an ISG54 minimal promoter in conjunction with five IFN-stimulated response elements, was used to prove the activation of the interferon regulatory factors (IRFs) pathway (Figure 5D).

### 3.6. THP-1 Monocytes Are Differentiated into Macrophage by Incubation with VHKs Conditioned Medium

**Figure 6:**
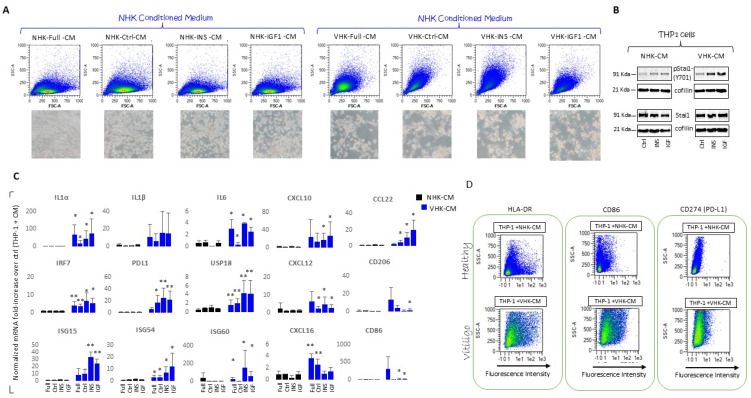
VHK-CM promotes THP-1 monocyte differentiation into macrophages, enhancing the pro-inflammatory cascade. (**A**) Dot plots and histograms showing differences in morphology and surface marker expression between NHK- and VHK-CMs treated THP-1 cells. Cells were initially gated using Forward Scatter Area (FSC) vs. Side Scatter Area (SSC) to discriminate cell size and intracellular structure complexity, respectively. The combined increased size and internal complexity argued for THP-1 differentiation into macrophage-like phenotypes. Representative pseudo-color dot plots are presented with the corresponding microscopic observation. (**B**) A representative Western blot analysis demonstrating the phosphorylating activation of Stat1 (pStat1-Y701) in THP-1 cells treated with VHK-CM. (**C**) mRNA analysis for the expression of interleukins, chemokines, and interferon-stimulated genes associated with monocyte activation. (**D**) Representative dot plots for CD86, HLA-DR, and CD274 staining. Normalized mRNA values are presented as fold-change ± SD over THP-1 treated with starved control cells in a CM. Note that * indicates *p* ≤ 0.05 and ** indicates *p* ≤ 0.01.

THP-1 cells, being monocytic cells capable of differentiating into macrophages and successfully polarizing into M1 and M2 types, also represent a physiologically relevant model to determine pro-inflammatory and anti-inflammatory properties after treatment [63]. Thus, we investigated the THP-1 differentiation profile in the presence of an IGF-1/Ins-conditioned secretome of the vitiligo keratinocytes. For this purpose, we primed the THP-1 cells with phorbol 12-myristate 13-acetate (PMA, 4 h 10 ng/mL) before exposure to a conditioned medium (CM) for 5 days (Figure 6A).

A light microscopy analysis displayed the loss of round single-cell morphology, and the acquisition of an adherent morphology characteristic of the macrophage-differentiated phenotype. Further, the light scatter measured by the forward scatter signal (FSC) and side scatter signal (SSC) parameters in cytofluorimetry reflected the modifications in size and granularity, respectively, confirming the cellular differentiation of the THP-1 cultures exposed to a vitiligo CM. The macrophage differentiation was additionally evidenced by the phosphorylation of the transcription factor Stat1 (Figure 6B). At this time point, the phenotype of the THP-1-derived macrophages was characterized by a gene expression analysis, the acquisition of different surface markers, and the pattern of cytokine production. The IL-1α, IL-1β, IL-6, IL-8, CXCL10, CXCL12, CXCL16, and PD-L1 were found to be upregulated in the THP-1 cells incubated with VHK-CM, with a specific further increase due to IGF-1 and Ins exposure (Figure 6C). Moreover, the IFN-induced transcriptional activation of three ISGs (ISG15, ISG54, and ISG60) was recorded as greatly augmented. Consistent with hyperactive type I interferon, an enhanced expression of USP18 (the major ISG15 isopeptidase) [65] was detected when the THP-1 cells were treated with a VHK supernatant. Further, at the mRNA level, we observed both M1 (CXCL10, IL-6, IL-1β, CD80, CD86) and M2 (CCL22, CCL18, CD163, CD206) differentiation markers (Figure 6D). Differentiation was validated using flow cytometry for a cluster of differentiation markers. The flow cytometry results displayed positivity for CD86 and HLA-DR (corresponding to the M1 pro-inflammatory type) as well as for CD206 and CD163 (corresponding to M2) confirming a heterogeneous and multi-functional response or a non-classic phenotypic pattern overlapping between the M1 and M2 phenotypes (Table 1).

## 4. Discussion

Despite the proven genetic association with several cutaneous and extra-cutaneous autoimmune diseases, the mechanisms involved in the initiation and development of auto-inflammation and autoimmunity in vitiligo patients are still debated. The epidemiological data reporting the comorbidity rates with diabetes mellitus are of particular interest since patients with vitiligo often exhibit systemic metabolic abnormalities such as an abnormal glucose metabolism, high fasting plasma, dyslipidemia, high blood pressure, an out-of-range C-peptide, and a low antioxidant capacity [32,33,37,38,39]. Consistently, the association of vitiligo with both type 1 and 2 diabetes reinforces the idea that both autoimmune and metabolic (non-autoimmune) components are involved in the pathogenesis of vitiligo. Recent clinical evidence argues for a link between systemic insulin resistance, generally asymptomatic in vitiligo patients, and disease onset [29,33,34]. However, the relationship between insulin resistance, defined as a defect in the insulin-mediated control of glucose metabolism, and vitiligo pathogenesis, having clinical manifestations confined to the skin, still lacks an explanation.

In the case of systemic insulin resistance, until pancreatic beta-cell activity no longer adequately meets the augmented insulin demand, extra glucose stays in the bloodstream due to decreased insulin-stimulated glucose transport/use and the cells suffer a hypoglycemic state (prediabetes) [66]. This condition resembles those observed in vitiligo cells since impaired intracellular metabolic activities result in the perception of ATP depletion like with hypoglycemia (even in the presence of euglycemia), requiring a supplemental import of glucose with a consequently undesired intracellular accumulation of glucose-related by-products. A similar scenario, consisting of reduced mitochondrial ATP synthesis that mimics the effect of a low glucose level, has been observed in neurons [67,68]. Accordingly, in vitro vitiligo cells manifest a bimodal weakness: in the presence of low glucose levels in the microenvironment ATP production is stunted, corresponding to a high level of pAMPK and autophagy chronic activation. These events, associated with enhanced Glut-4 expression and the augmented import of glucose, supply the energetic requirement, functioning as a metabolic adaptation. However, the consequent cytoplasmatic hyperglycemia causes an overactive mTOR/S6, leading to a negative feedback loop in the IGFR/InsR intracellular signaling (IR at cellular level). In this case, IGF-1 and Ins signaling are attenuated by chronic IRS1 inhibitory phosphorylation and the related mitogenic stimulation is whittled down. Even if reduced, the stimulation of anabolic activities due to IGF-1/Ins contributes to an aggravation of the energetic imbalance (further ATP depletion) and oxidative stress (an increase in ROS). Differently, in a hyperglycemic condition, the high glucose availability in the medium coupled with the hyperfunctional glucose imported into vitiligo cells, facilitates its intracellular accumulation as demonstrated by the excessive dislocation of non-enzymatically derived glycated by-products in the surrounding microenvironment. In this instance, the capacity to respond to mitogenic stimulations, such as Ins and IGF-1, is recovered, but the intensified mTOR/S6 phosphorylation reinforces the negative feedback leading to signaling desensibilization confirmed by low AKT phosphorylation. The AKT pathway plays an important role in response to insulin and represents protection against hyperglycemia [68]. An impairment of AKT phosphorylation renders keratinocytes and melanocytes more susceptible to ROS-dependent apoptosis [69]. In addition, high glucose levels have other effects on keratinocyte physiology that could lead to abnormal functions of the skin. In high-glucose environments, keratinocyte differentiation is markedly slowed [69,70]. Sugar-treated keratinocytes also display premature senescence [71]. Accordingly, diabetes mellitus (both type 1 and type 2) patients carrying chronic defective insulin signaling in the keratinocytes often suffer non-healing wounds and foot ulcers [50,72]. Similarly, vitiligo patients may have defective epidermal permeability barrier functions [73]. Alterations in keratinocyte differentiation and stratum corneum lipid profiles in non-lesional skin have been recently reported [34].

Interestingly, although persistent activation of AMPK is a common trait of vitiligo cells, there was also a modest but recurrent upregulation of mTOR and *p*-mTOR even though AMPK is a known inhibitor of mTOR. Normally, when mTOR is inactivated, S6 phosphorylation is depressed. Here, in vitiligo cells, the lack of coordination between AMPK and mTOR/S6 signaling, the two most important metabolic signaling pathways, reflects the improper perception of hypoglycemia. S6 phosphorylation and the levels of pAKT were more pronounced in vitiligo keratinocytes. A possible explanation might be the intense metabolic activity of keratinocytes. Interestingly, the dysfunctional AKT/mTOR pathways associated with enhanced glucose metabolism is considered important factors linking the psoriatic keratinocyte phenotype to metabolic syndromes [74]. A correlation between increased intracellular ROS, a diminished ATP level, and mitochondrial hyperpolarization has been reported in other chronic inflammatory diseases such as systemic lupus erythematosus as a part of T cells’ hyper-function [75]. More interestingly, in addition to lymphocytes and monocytes, the keratinocytes of diabetes patients also demonstrated greater membrane potential, delineating a similar pattern of mitochondrial dysfunction [58]. Interestingly, in pancreatic β-cells mitochondrial hyperpolarization is induced by endoplasmic reticulum stress and prevented by insulin stimulation [76]. AMPK activation promotes catabolic processes and stimulates glucose uptake as demonstrated by the LC3 II level and the reduced glucose in the medium, respectively. However, mTOR is also a sensor of intracellular energy levels and in some specific conditions, mTOR might promote glucose uptake and the metabolic reprogramming named the Warburg effect, characterized by the prevalent use of the glycolytic pathway to provide energy.

At the same time, because of the difficulty of managing mitochondrial metabolic activity, the impaired ATP production and high glucose availability finally led to glucotoxicity and inflammation. Accordingly, the number of AGEs, markers glucose metabolism impairment, detected in the vitiligo cells were higher compared to the healthy cells. Consistent with this finding, we recently reported abnormally high concentrations of AGEs in the plasma of vitiligo patients [34]. Nevertheless, a dependence on cell type is clear, since melanocytes display an opposite feature concerning ROS production in the context of glucotoxicity. The failure of the hyperglycemic state to recover proliferative stimulation in VHMs corresponds to the absence of an overexpression of Glut4 in these types of cells, further indicating that intracellular glucose concentration is the limiting factor for vitiligo wellness. Meanwhile, our data showed a global limited capacity of melanocytes to be stimulated by Ins and IGF-1. Interestingly, higher differences in intracellular ATP content were observed in melanocytes. A possible explanation might reside in the intense metabolic coordinated network required for melanogenesis and its efficient stimulation by hormonal treatment.

Glucose-mediated insulin resistance provides a feedback mechanism sometimes independent of the activation of mTOR and it is due to direct phosphorylation and inhibition of IRS-1 by S6 kinase [77]. Overall, for the first time, our data definitively prove insulin resistance at a cellular level in vitiligo cells. However, in an insulin-resistant state, a compensatory increase in the membrane pool of Glut4 is considered a required mechanism for adequate glucose uptake. By contrast, in vitiligo cells the mechanism appears inverse: the enhanced import of glucose due to mitochondrial inefficiency provokes chronic mTOR-S6 signal amplification and IRS1 phosphorylation feedback, causing insulin resistance. The fact that IR in skin-derived vitiligo cells is manifested long after biopsies have been removed from the patients, suggests that the alterations related to glucose metabolism are intrinsic and cannot be justified by the action of systemic factors such as a prediabetic condition.

Even though all cell types derived from vitiligo non-lesional skin manifested an energetic disequilibrium, the functional consequences of this state appear amplified in the keratinocyte lineage. Specifically, VHKs are more prone to assume a pro-inflammatory feature. As the most dominant cell type in the skin, keratinocytes play a prominent role in skin immunity. Of note, because of the physiological organization of the epidermal melanin unit, melanocytes are fully surrounded by keratinocytes, and it is retained that keratinocytes play a central role in vitiligo. We demonstrated that VHK’s impairment of intracellular metabolic activities initiates a severe inflammatory state converging in the production of second messengers important in innate immunity activation, as evidenced by THP-1 monocyte differentiation, and possibly results in further amplification of the immunity network. Interestingly, at the molecular level, we observed a strong increase in the number of inflammatory mediators and interferon-stimulated genes (ISG15, ISG54, and ISG60) when the supernatant used was derived from VHKs exposed to Ins and IGF-1, confirming the important role of the associated signaling in vitiligo pathogenesis. The overexpression of USP15 has been previously reported in vitiligo skin tissues as well as in the blood of patients with vitiligo [78]. An atypical innate immune response is a significant reason for the breakdown of autoimmune tolerance, which is closely related to the development of autoimmune diseases [79]. According to previous studies, both prolonged M1 activation as well as altered M2 function can contribute to the onset and activity of autoimmune disorders. Flow cytometry for the cluster differentiation of markers evidenced M1-like and M2-like polarization when THP-1 monocytes were exposed to a VHK-conditioned medium. Further, regarding autoimmunity and inflammation, the view of pro-inflammatory M1 macrophages and M2 macrophages suppressing inflammation seems to be an over-simplification because these cells display a marked plasticity and represent a large scale of different immunophenotypes with overlapping properties. In autoimmunity, the disparity of the M1/M2 macrophage phenotype balance because of the protracted activation of M1 macrophages or the altered function of anti-inflammatory M2 macrophages can trigger and further promote inflammation [80,81]. Sain and collaborators reported an incremental increase in either M1 and M2 macrophages and an elevated M1/M2 ratio in the blood and per-lesional skin of vitiligo patients [82]. The contemporary presence of activated M1 (releasing cytokines and chemokines) and M2 macrophages in vitiligo patients (required for tissue repair and reconstruction) might be explained by the inflammation nature of the disease (immune destruction of melanocytes) and the possible endless attempt to replace melanocytes in a setting of the intrinsically compromised regenerative capacity of pathological tissues. Overall, from the functional point of view, THP-1 cells primed with PMA and exposed to a VHK-CM, particularly those treated with IGF-1 and Ins, produced a large amount of immunostimulatory molecules which amplify inflammation.

## 5. Conclusions

Mitochondria-impaired activity strongly impacts vitiligo cells’ energetic capacity leading to reduced ATP production. These disruptions may produce a precarious biological milieu that disrupts insulin signaling, a fact that, in consideration of several epidemiological data, convinces us to associate vitiligo with metabolic diseases. Here, we provided evidence of Ins/IGF-1 resistance at the cellular level involving the dermal and epidermal non-lesional cells of vitiligo patients. Keratinocytes appeared to be particularly sensitive to glucotoxicity, as evidenced by the marked rise in ROS, AGEs, and pro-inflammatory molecules in presence of Ins/IGF-1 stimulation. A possible explanation might reside in the intense proliferative rate of this type of cell, ensuring the continuous physiological turnover necessary for epidermis renewal and the necessity of intense anabolic activities to produce several proteins and lipids forming the stratum corneum. Importantly, keratinocytes orchestrate a major process involved in inflammatory and autoimmune skin diseases.

The definition of the metabolic imprint of inflammation in vitiligo has clinical implications and opens a new translational research perspective. Based on this study, it is possible to develop conceptually new treatments, inspired by diabetic treatments such as an insulin sensitizer.

## Figures and Tables

**Table 1:** Percentage of THP-1 cells expressing M1 and M2 macrophage differentiation markers.

Marker	NHK Full	NHK st	NHK Ins	NHK IGF-1	VHK Full	VHK st	VHK Ins	VHK IGF-1
**CD86**	8.0 ± 8	4.7 ± 1	4.0 ± 1	4.7 ± 2	29.3 ± 13	25.0 ± 20	24.0 ± 25	31.0 ± 22
**HLA-DR**	36.0 ± 9	33.5 ± 6	32.0 ± 5	37.7 ± 2	51.5 ± 8	61.7 ± 16	55.75 ± 12	53.5 ± 17
**CD206**	20.5 ± 15	6.5 ± 1	4.0 ± 1	6.0 ± 1	47.0 ± 17	33.5 ± 14	32.5 ± 18	32.0 ± 20
**CD163**	21.0 ± 20	3.0 ± 2	2.0 ± 1	3.0 ± 2	40.0 ± 6	12.0 ± 1	11.5 ± 2	11.0 ± 6

## Data Availability

The data supporting the findings of this study are available from the corresponding author upon reasonable request.

## Supplementary Materials

The following supporting information can be downloaded at: https://www.mdpi.com/article/10.3390/cells14080565/s1, Table S1: Demographic characteristics of patients and healthy donors; Figure S1: Epidermal and dermal proliferation is attenuated by high glucose availability; Figure S2: Glucose availability impacts on the phosphorylation of intracellular components og Ins/IGF-1 pathway; Figure S3: Alteration of metabolic markers in vitiligo cells.
