# Supplementary figures and images for: Defective Intracellular Insulin/IGF-1 Signaling Elucidates the Link Between Metabolic Defect and Autoimmunity in Vitiligo
Source: Cells. 2025 Apr 9;14(8):565. doi: 10.3390/cells14080565 (PMC12025416; doi:10.3390/cells14080565)

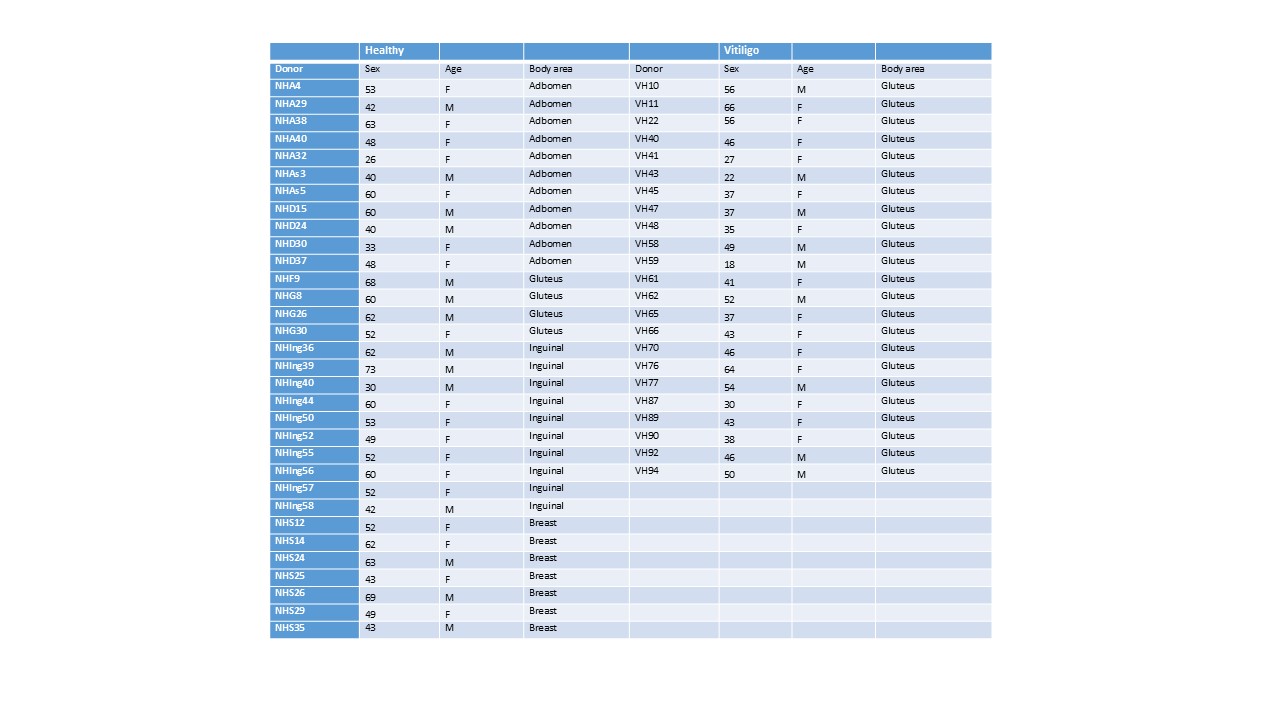

Supplement: Supplementary file 1 [file cells-14-00565-s001.zip › Suppl. Figures Caputo et al/Diapositiva1.JPG]

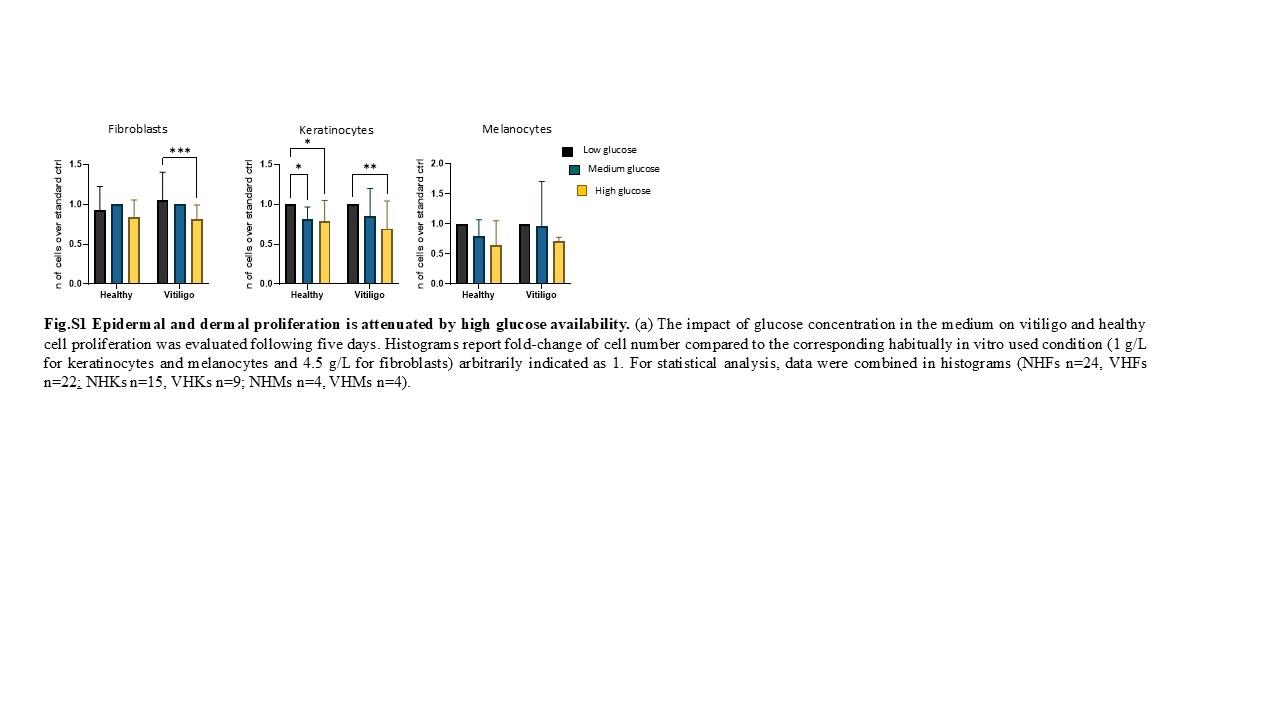

Supplement: Supplementary file 1 [file cells-14-00565-s001.zip › Suppl. Figures Caputo et al/Diapositiva2.JPG]

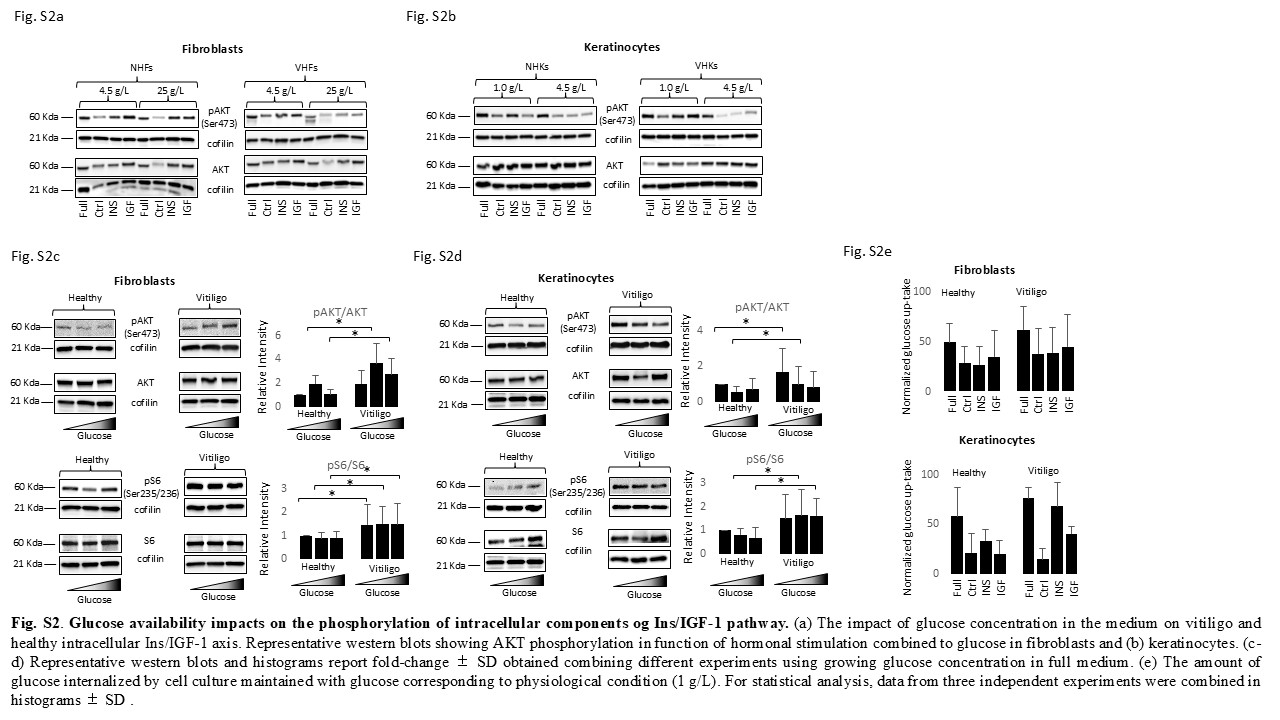

Supplement: Supplementary file 1 [file cells-14-00565-s001.zip › Suppl. Figures Caputo et al/Diapositiva3.JPG]

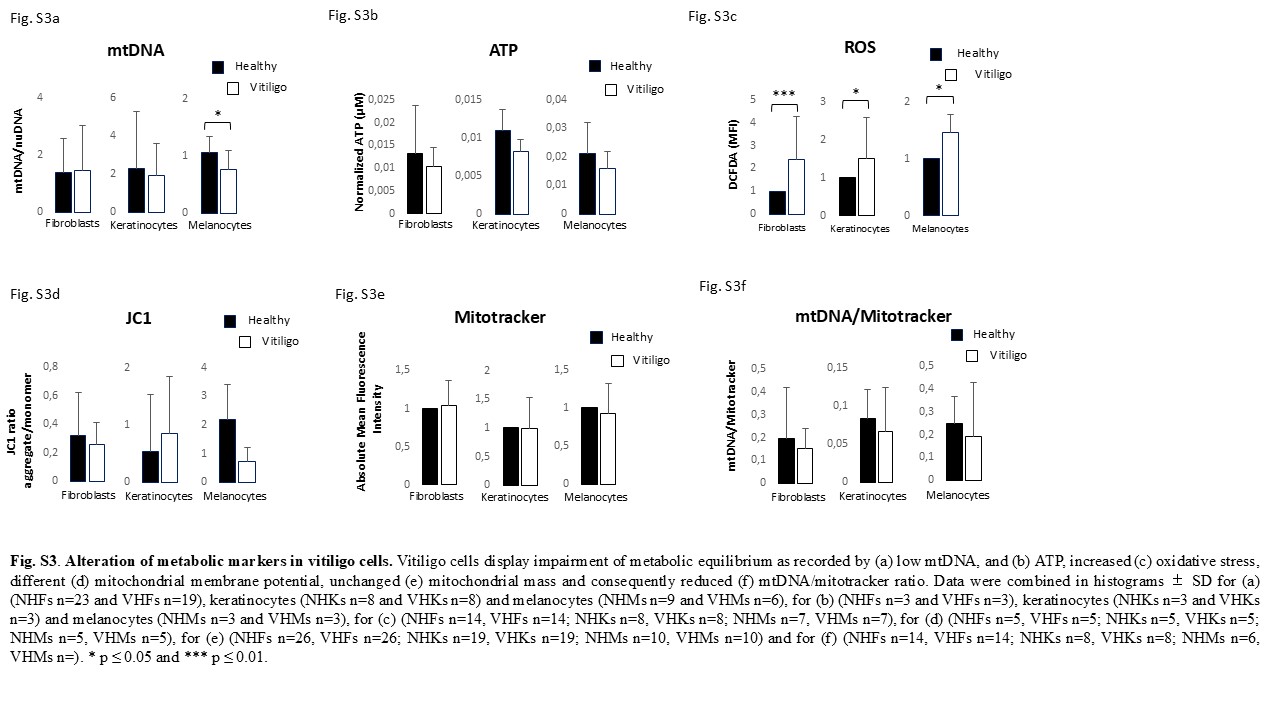

Supplement: Supplementary file 1 [file cells-14-00565-s001.zip › Suppl. Figures Caputo et al/Diapositiva4.JPG]
